# Supplementary figures and images for: Predicting biomass of rice with intermediate traits: Modeling method combining crop growth models and genomic prediction models
Source: PLoS One. 2020 Jun 19;15(6):e0233951. doi: 10.1371/journal.pone.0233951 (PMC7304626; doi:10.1371/journal.pone.0233951)

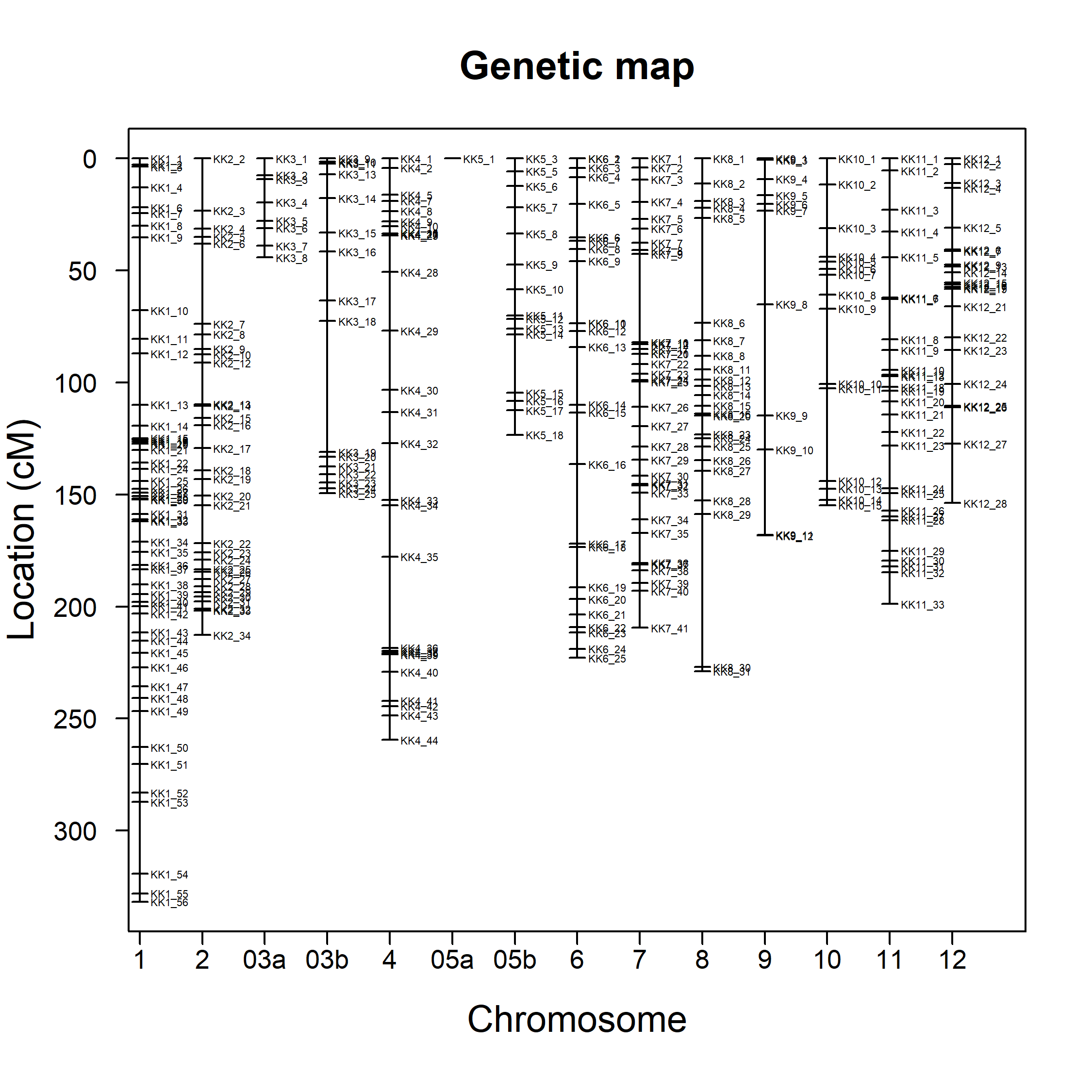

Supplement: S1 Fig — (TIF) [file pone.0233951.s001.tif]

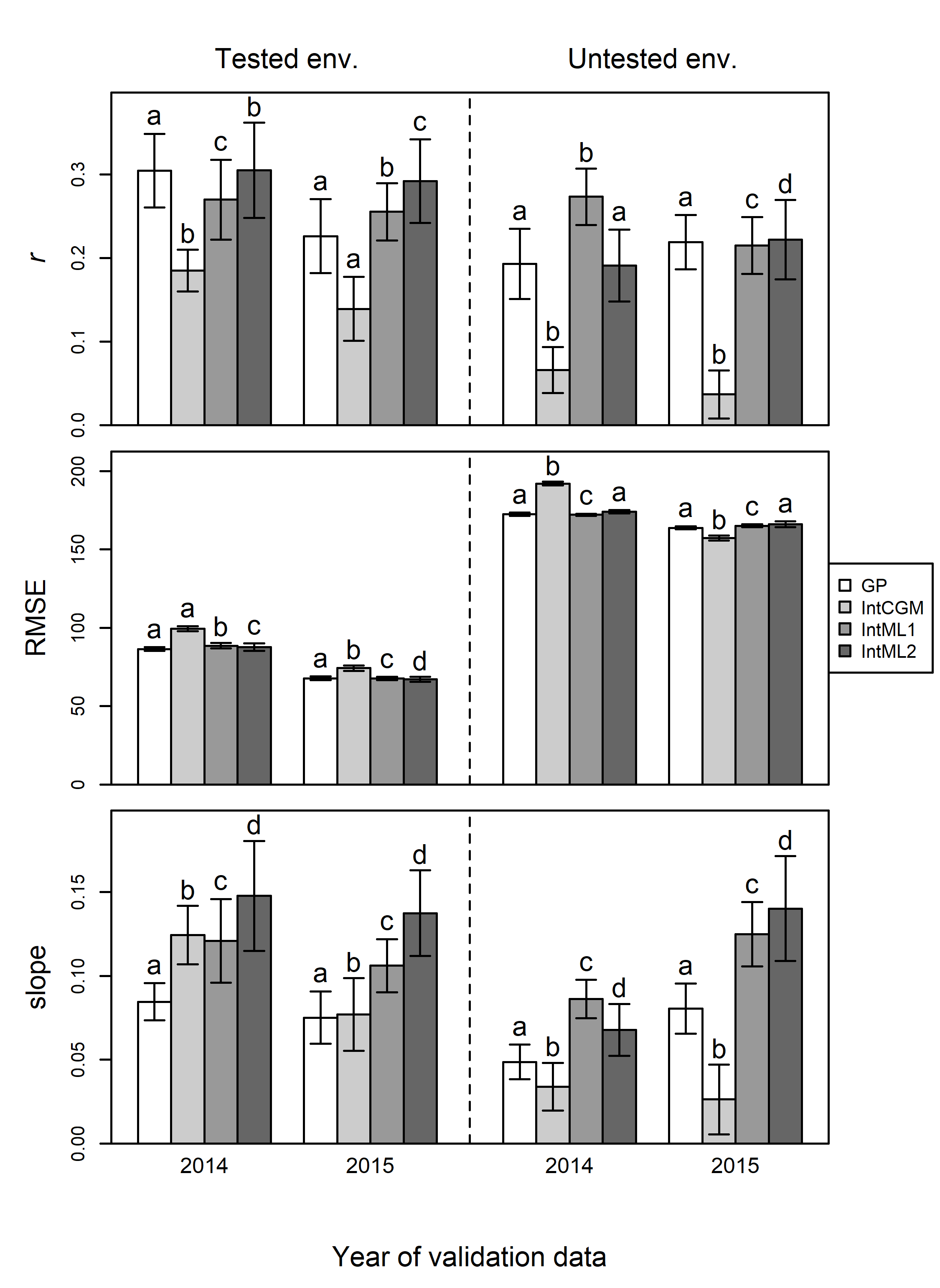

Supplement: S2 Fig — The result of prediction of tested (left) and untested (right) environments are shown. LASSO was chosen as a representative GP model. Three indices were used: Correlation coefficient (r), RMSE (root mean squared error), and slope of the regression line for predicted and observed values. Error bars represent ± 1 s.d. Letters above the bars indicate significant differences determined using the Steel–Dwass test (p < 0.01). (TIFF) [file pone.0233951.s002.tiff]
