# Supplementary material for: Predicting biomass of rice with intermediate traits: Modeling method combining crop growth models and genomic prediction models
Source: PLoS One. 2020 Jun 19;15(6):e0233951. doi: 10.1371/journal.pone.0233951 (PMC7304626; doi:10.1371/journal.pone.0233951)
Supplement: Attachment — Submitted filename: ResponseToReviewers.docx [file pone.0233951.s007.docx]

Graduate School of Agricultural and Environmental Biology, The University of Tokyo
1-1-1 Yayoi, Bunkyo, Tokyo 113-8657
Tel.: 81-3-5841-5068

E-mail: aiwata@mail.ecc.u-tokyo.ac.jp

August 3, 2019

Dr. Joerg Heber

Editor-in-Chief, PLOS ONE

Dear Dr. Heber,

Re: PONE-D-18-34549 “Predicting G×E in biomass of rice: modeling method combining crop growth models and genomic prediction models”

We would like to resubmit our revised manuscript. According to the points raised by the reviewers, we revised the manuscript as described in the following pages.

We thank you in advance for considering the revised manuscript for publication in *PLoS ONE* and look forward to hearing from you at your earliest convenience.

Yours Sincerely,

Hiroyoshi Iwata, Ph. D

Department of Agricultural and Environmental Biology

Graduate School of Agriculture and Life Science

The University of Tokyo

1-1-1 Yayoi, Bunkyo, Tokyo, Japan

Tel: +81-3-5841-5069

Email: aiwata@mail.ecc.u-tokyo.ac.jp

**Reply to the reviewers**

**Reviewer #4: In this manuscript, the authors tried to integrate the physiological model into GP to improve prediction accuracy for biomass in rice. However, I have two major concerns.**

**(1) The authors may have misused the concepts of environmental effect and G×E interaction. I think the authors tried to include the environmental effects into GP, rather than G×E interaction. If two genotypes grow slower in one environment, but faster in the other environment, we say the environment has an effect on the growth. If genotype A performs better than genotype B in one environment, but poor in the other environment, we say G×E interaction is present. Environmental effect and G×E interaction are two different concepts.**

In our study, we took GxE rather than E into account to explain the phenotypes of analyzed and validated genotypes (cultivars/lines). The IntCGM model, which we used in the study, considered the G×E interaction. For example, The effect of temperature on biomass can differ between genotypes because the intermediate traits, such as final leaf age and growth rate of the number of leaf age, differ among genotypes. Please consider one genotype A with a larger final leaf age and genotype B with a larger growth rate of leaf age. When they are cultivated in an environment with lower temperature, genotype B will grow larger because they can get more leaves with a higher growth rate. On the other hand, when they are cultivated in an environment with higher temperature, genotype A will grow larger because genotype A can reach to the final leaf age and can have larger biomass and yield due to its larger final leaf age. We have to note that even the change of ranking in biomass or yield will occur if intermediate traits have a large variety.

**(2) I missed the authors’ major objective to consider the physiological model into GP. Was the objective for the breeding purpose, or for agricultural production? If for breeding, biomass is normally not a breeding-targeted trait.**

The objective of this research is for the application for breeding. We partly understood the reviewer’s comment, but think that biomass is also an important component for the improvement of yield potential in rice. Especially when heading date becomes earlier, plants cannot have sufficient vegetative growth and have smaller biomass so that yield potential decreases. Also in a crop growth model, it is common to express the yield as the multiplication of final biomass and a harvest index. In this study, we also tried to predict panicle weight, but the prediction accuracy cannot be improved (L469-474). Although the result for the prediction of panicle weight was not so successful, we think our results can have good suggestions and information for the genetic improvement of yield potential in rice.

**It should also be noted that non-repeatable environmental effects can hardly be utilized in breeding.**

It is true that the non-repeatable environmental effects are currently ignored in breeding. But consideration of such non-repeatable effects will benefit to the crop breeding. If environmental effects can be estimated, for example, we can consider variability of yield performance of one genotype over varous environments by using historical environmental data. Such information will help breeders to select stable genotypes for the target environment. We added an explanation in the discussion part (L496-499).

**Some specific comments are:**

**(1) Introduction: This part was too much focused on the theoretical aspects of GP. Breeding is an applied science. A number of application studies have been published on the use of GP in animal/plant breeding (e.g. parental selection, cross prediction etc.), which should also be covered.**

We added some references for GP in animal breeding [2] and plant breeding [3-5] and in the introduction section (L51).

**(2) L137-L145: It’s not clear how the genetic map was constructed; how the map information was used in prediction, or can help the prediction.**

Only SNPs data were used in the prediction, and map information, such as distances between loci, was not used. We provided a genetic map in the manuscript to show it as the basic information of genotype data. Because it might be a bit confusing that the information unused in the analysis directly was shown in the manuscript, we made Figure 1 as a Supplemental Figure (S1).

**(3) L157: It’s not clear what is the relationship between the 112 cultivars and the RIL population.**

Because the RIL population was cultivated in only one field, it was difficult to estimate crop model parameters for heading date. To obtain the model parameters, we used heading dates recorded in METs with 112 rice cultivars, including Kinmaze and Koshihikari. Most of the 112 cultivars were developed in Japan. We added an explanation about this point in L156-157. Also, we added supporting information (S2 Table) where the names of 112 cultivars were recorded.

**By the way, the 112 cultivars should be introduced in “Plant materials” in MM.**

The 112 cultivars had been already introduced in L157-161.

**(4) L163-L168: ANOVA was mentioned here, but I didn’t see any ANOVA table in results. If G×E interaction is the major target, any results on G×E interaction should be provided in details.**

As you mentioned, we added an ANOVA table in the supporting information (S3 Table).

**(5) L169-L172: Need re-writing. It’s not clear which two methods belong to Gaussian process regression.**

We changed the description of regression methods as “For building the GP models, we employed four methods. Two of them were regularized regression: ridge regression (RR) and LASSO, and the other two were Gaussian process regression (reviewed by [35]): one based on an additive relationship matrix (GBLUP) and the other on a Gaussian kernel matrix (RKHS) as a representative of covariance matrix” (L170-174).

**I noted one previous reviewer also raised the issue one GP models used in this study. The manuscript needs better organization to increase the readability. The author may want to focus just one model, and move other models as supplementary.**

We introduced four prediction models in our study because using LASSO as representative of GP model is not very common. In general, GBLUP is the most popular method for GP, but LASSO performed best in our case.

**(6) L311: The correlation between predicted and observed is the best one to measure the prediction accuracy. No need to consider the other two.**

As the reviewer suggested, correlation between predicted an obaserved values is one of important measures of the prediction accuracy. We think different two indices are also important and the reason why we think they are important is described in the manuscript (L443-457).

**(7) Some parts in MM reads like results, for example Figure 3.**

As you mentioned, we moved Figure 3 to the result part. A part of the manuscript (L322-323) was modified. Figure 3 and 4 in the original manuscript were renumbered as Figures 3 and 2 in the revised manuscript.

**(8) Fig. 1: In current format, the map is less informative. At least maker names should be added.**

We added temporal names of the SNP markers in Fig. 1 (S1 Figure in revised manuscript). Also, we uploaded a table as supporting information (S2 Table) in which formal marker names, physical and recombinant distances are included.

**Some gaps may be over 50 cM. I have some doubt on the quality of the map.**

As you mentioned, some markers have long distances between them. All the SNPs we used have been located to the reference sequence of Nipponbare and linkage groups of map were not derived from the segregation information. Thus, miss grouping because of segregation bias may not be occurred. The long gap between markers is due to the absence of polymorphisms between two parents, because we selected the two parents from a Japanese cultivar group which have relatively small genetic variations.
