# Supplementary material for: Predicting biomass of rice with intermediate traits: Modeling method combining crop growth models and genomic prediction models
Source: PLoS One. 2020 Jun 19;15(6):e0233951. doi: 10.1371/journal.pone.0233951 (PMC7304626; doi:10.1371/journal.pone.0233951)
Supplement: Attachment — Submitted filename: ResponseToReviewers_20200202.docx [file pone.0233951.s008.docx]

Graduate School of Agricultural and Environmental Biology, The University of Tokyo
1-1-1 Yayoi, Bunkyo, Tokyo 113-8657
Tel.: 81-3-5841-5068

E-mail: aiwata@mail.ecc.u-tokyo.ac.jp

February 1, 2020

Dr. Joerg Heber

Editor-in-Chief, PLOS ONE

Dear Dr. Heber,

Re: PONE-D-19-23130 “Predicting G×E in biomass of rice: modeling method combining crop growth models and genomic prediction models”

We would like to resubmit our revised manuscript. According to the points raised by the reviewers, we revised the manuscript as described in the following pages.

We thank you in advance for considering the revised manuscript for publication in *PLoS ONE* and look forward to hearing from you at your earliest convenience.

Yours Sincerely,

Hiroyoshi Iwata, Ph. D

Department of Agricultural and Environmental Biology

Graduate School of Agriculture and Life Science

The University of Tokyo

1-1-1 Yayoi, Bunkyo, Tokyo, Japan

Tel: +81-3-5841-5069

Email: aiwata@mail.ecc.u-tokyo.ac.jp

**My major concern was with the clarity of the work. The major contribution is the combination of genomic selection and a crop growth model to predict biomass. The approach is quite complex (Figure 2), but the logic is straightforward. Biomass is very strongly correlated with leaf area accumulated prior to flowering time. Both growth rate and flowering time have genetic components. Thus, genomic prediction can predict differences in biomass through these "intermediate traits." The key point seems to be that direct genomic prediction of biomass is worse (though not that much worse) than the integration the crop growth models. The discussion would benefit from expanding on why using the growth models usually leads to better predictions.**

The improvement in the prediction accuracy of our model was partly caused by the prediction of heading date, which was included in both IntCGM and IntML. Because we considered the interaction between genotypes on one hand and environmental factors the other, the predicted values of heading date as intermediate traits can contribute to explain the pattern of G×E in biomass. We added the related descriptions to explain the reason why the new models (IntCGM and IntML) performed better than GP (L388-393). Another benefit to use IntCGM has been described in a later part (L425-450).

**I would also include the analysis of yield mentioned in the discussion in results**.

As you suggested, we moved the description about the prediction of panicle weight (L470-L474 in old manuscript) to the result part (L370-373). Also, we provide S2 Figure to describe the prediction accuracy of panicle weight (The figure caption was added in L733-738).

**I was surprised that r of the predicted values vs untested environment values were so high (Figure 6). It seems the model trains on the means/ lsmeans from two very different replicates in each year. Perhaps this approach is leading to high correlations. How does the model perform if predictions are tested across the single replicates in the following year? This work would be more likely used in this context it seems.**

We added the table of narrow-sense heritability calculated by replicates (S4 Table). We, however, did not test performances of the models when they are trained with a single replicate, but it is clear that the prediction accuracy of all the models decreases at a certain level. Because the replicates were cultivated in the same environment (at least same in environmental variables used in this study), the differences in phenotypic values between the replicates cannot be explained with either genotype data or environmental data. Thus, the separate modelling with a single replicate will only enlarge noises to use a fewer number of replicates for training models, and thus leads to decreases in the prediction accuracy for all the models.

**The rationale for the other work reported in the manuscript was unclear to me. The discussion first describes how rice growth differed between 2014 and 2015. I'd omit this.**

As you suggested, we removed the paragraph “Comparison of crop traits between different temperature patterns” (L378-385 in original manuscript).

**The discussion then discusses Figure 6 which has the results of the growth model/genomic prediction. If the other reported data made a scientific contribution, I would discuss them more fully or remove them.**

We described about three indices (*r*, RMSE and slope) in the paragraph (L388-400 in original manuscript). We removed the notation about the slope because it was duplicated with a later paragraph (L436-450). Also, we removed the notation about RMSE (L396-398 in original manuscript). The RMSE of IntCGM in the prediction under the untested environment showed different tendencies in the prediction in the untested environment (Fig 6), but the reason was due to the specific structure of crop growth model we used. Because the explanation for the reason will be so long but have a little generality for other studies, we decided to remove the description. Finally, the description about *r* was shortened and moved to L384-388, before a newly added part describing the reason why our integrated models could improve prediction accuracies (L388-393). We believe that this revision made the aim of this paragraph clearer.

**Reviewer #1: 2019-11-19**

**I have read the revised manuscript and authors’ responses to reviewer’s comments, and thank that the manuscript is well prepared and the responses are almost adequate. I have two questions and would like the authors to address in their Discussion section.**

**The first is related to the reviewer 4’s question on how to deal with non-repeatable genotype by environment interaction (GE). This is a very good question for any study to be useful for breeding. The authors response was to use “historical environmental data or simulated (assumed) future environmental data.” I think this is a very important point and needs to be emphasized if their models are to be used in assisting breeding.**

**Indeed, GE has always been a challenge to plant breeding, and it is important to differentiate repeatable vs. non-repeatable GE and deal with them differently. In plant breeding, non-repeatable GE is dealt with by testing at multiple locations for multiple years so as to represent the population of environments in the target environment (Yan, 2016 Crop Science). For reliable genomic prediction (GP), genomic models must also be developed based on phenotypic data from such multi-location multi-year trials (Yan et al., 2019, Crop Breeding, genetics, and Genomics). This also applies to prediction models that integrate GP and physiological models.**

As you suggested, we viewed our result from the aspect of the decomposition of G×E. We assume that non-repeatable G×E can decompose G×E in variations explained by environmental factors and ones unexplained by environmental factors; the former and latter are referred to as explainable and non-explainable G×E, respectively. The explainable G×E was because of differences in environments between years, whereas the latter was not explained by any obvious environmental factors, therefore, treated as noise. Improvement of prediction accuracies of models in the tested environment indicate that the integrated models can predict the explainable G×E variations. Furthermore, the improvement of accuracies in the untested environment indicate that the integrated models can predict the repeatable G×E variations, because difference between the field trials in two years, where the patterns of temperature and day length differed, was as large as those between locations.

We added a discussion about this topic (L486-503) with a reference you noted [64] (L728-729).

**Furthermore, models integrating multi-location multi-year data have to be validated by similarly obtained phenotypic data. It remains a question whether an “integrated” model actually predicted better than GP alone.**

Indeed, the performances of our integrated models were not tested with multi-location data. However, as emphasized in the method part (L103-109), sowing and transplanting were performed in different months between years to produce results under different conditions of day length and temperature. Because of different cultivation periods during 2014 and 2015, the 2-year experiments were not simply yearly replications but were expected to induce different growth patterns under different environmental conditions. Thus, as shown in the better prediction accuracies in the untested environment than GP (Fig 6), it is certain that our models predicted better in the prediction under different patterns of day length and temperature.

**My second question/comment is how far does the crop simulation models have to go? In the current study, leaf number, tiller number, and leaf size were simulated using daily temperature and day length to simulate biomass. However, the complexity of the physiological and biological process leading to biomass or grain yield is unlimited. For example, should we go by hourly temperature? Should we also consider CO2, water, and mineral nutrient availability in the models? Is there an optimum level of complexity to target in integrated models?**

As we wrote in the discussion part (L466-468), it should be avoided to use a crop growth model with too much complexity. However, it is an important problem to find an optimum model with an appropriate number of parameters. We should omit unnecessary variables depending on the target dataset. A sensitivity analysis will be effective to select modules of the models in which variables with little influence on target traits will be distinguished. We added a description of that point (L468-471).
