# Supplementary material for: Predicting biomass of rice with intermediate traits: Modeling method combining crop growth models and genomic prediction models
Source: PLoS One. 2020 Jun 19;15(6):e0233951. doi: 10.1371/journal.pone.0233951 (PMC7304626; doi:10.1371/journal.pone.0233951)
Supplement: Attachment — Submitted filename: ResponseToReviewers_20200416.docx [file pone.0233951.s009.docx]

Graduate School of Agricultural and Environmental Biology, The University of Tokyo
1-1-1 Yayoi, Bunkyo, Tokyo 113-8657
Tel.: 81-3-5841-5068

E-mail: aiwata@mail.ecc.u-tokyo.ac.jp

April 19, 2020

Dr. Joerg Heber

Editor-in-Chief, PLOS ONE

Dear Dr. Heber,

PONE-D-19-23130-R1 “Predicting G×E in biomass of rice: modeling method combining crop growth models and genomic prediction models”

We would like to resubmit our revised manuscript. According to the points raised by the editor, we revised the manuscript as described in the following pages.

Also, we offer to change the title to “Predicting biomass of rice with intermediate traits: modeling method combining crop growth models and genomic prediction models.” It is a revise to meet the editor’s proposal in which prediction of G×E is not considered as a suitable expression to represent our research.

We thank you in advance for considering the revised manuscript for publication in *PLoS ONE* and look forward to hearing from you at your earliest convenience.

Yours Sincerely,

Hiroyoshi Iwata, Ph. D

Department of Agricultural and Environmental Biology

Graduate School of Agriculture and Life Science

The University of Tokyo

1-1-1 Yayoi, Bunkyo, Tokyo, Japan

Tel: +81-3-5841-5069

Email: aiwata@mail.ecc.u-tokyo.ac.jp

**My major concern remains with the clarity of the work. For example, a reader should be able to move from introduction to results and get a sense of the findings. The questions posed in the introduction should specifically addressed in results. Now, results is mostly composed of technical statements. Re-writing the section to contain paragraphs with topic sentences would help improve clarity. The article will have greater impact if it can be readily understood. Specific lines to improve are listed below but please edit broadly.**

As you pointed out, we revised our manuscript in the following points.

L71-79: We clarified what was remained to be improved in recent studies of the integrated models of GP and CGM.

L80-90, L97-116: We stated about the originality of our research following to the description of use of growth-related traits (L80-90). This revision enabled readers to easily understand why we chose two-step approach. Also, we modified later paragraphs to take consistency of the manuscript (L97-116).

L196: We moved a description of 10-fold cross-validation (L344 in the original manuscript), which should be placed in the material and methods part.

L349-351, L352-L353, and L374-376: We added explanations about topics of each paragraph for whom move to the result part from the introduction part directly.

L378-379: We added a necessary explanation about the result.

L381: We changed the expression for easy understandings.

L400-404: We re-written the paragraph to make the result clearer.

L470-471: We added a description about recent studies of multi-trait GP to clarify the improvement of our study.

**A central point of the article is that the inclusion of growth-related traits into models enabled G x E effects to be predicted. G X E indicates variation in genotypic responses to environment variation.** **In this study, it seems genotypes were analyzed in single environments, so there is no variation due to the environment except random error.** **Within this single environment, some genotypes grow faster and others slower, so genotypes will have different growth rates relative to environmental factors such as temperature, but I would refer to these genotypic differences as genetic effects not G x E.** **G x E effects could explain differences in genotypes between the two years of the study, but this aspect does not seem to be the focus.** **Specific text includes lines 38-41; 388-390; 391-393, and elsewhere such as 483. Please clarify.**

As you pointed out, we edited our manuscript in the following points.

L26-30, L38-39: We changed expressions in the abstract to take consistency with the following revisions.

L55-56, L59-60, L62-63, and L66-67: We changed expressions of explanations about recent researches in the introduction part to make it correspond with revisions in the discussion part.

L420-426: We discussed the improvement of the prediction accuracy in IntCGM and IntML from an aspect of the use of the intermediate traits, not from an aspect of prediction of G×E.

L536-543: We placed a paragraph to discuss the future possibility of prediction of G×E with IntCGM, instead of the paragraph in which we argued that IntCGM succeeded in the prediction of G×E.

Also, we offer to change the title to “Predicting biomass of rice with intermediate traits: modeling method combining crop growth models and genomic prediction models” and running title to “Predicting biomass of rice with intermediate traits” (L1-4). We removed the expression of prediction of G×E from them.

**In previous comments, I wrote, “I was surprised that r of the predicted values vs untested environment values were so high (Figure 6).” My understanding is that genomic prediction accuracies estimated for 2014 lines using 2014 data (“tested”) were less accurate than genomic prediction models trained with 2014 data and used to predict 2015 (“untested”) (Figure 6). This odd result, if correct, still does not seem to be explained.**

The result is correct. We added a description about this result (L427-442) and two references (L751-756). Also, we modified figure labels of Fig 6 to make it easier to understand which data was used as training or test data. Corresponded revisions in manuscript are in L395, L401, L410.

This intuitively unexpected result might be owing to two reasons. One is the low heritability of biomass in 2014, which led to lower prediction accuracy in the models [58-59]. To reduce the influence of the heritability level on the index of the prediction accuracy (i.e., a correlation coefficient between observed and predicted phenotypes), the value of *r* was adjusted by dividing it by the square root of genomic heritability. The adjusted values of *r* became 0.652 and 0.746 for the biomass in 2014 and 2015, respectively, and had smaller differences than the previous *r*.

Another reason for the higher biomass prediction accuracy in 2015 is the GS model built with LASSO. In Fig 5, the biomass prediction accuracy was lower in LASSO than in other models in 2014, whereas the result was the opposite in 2015. Polygenic marker effects seemed more dominant in biomass in 2014 than in 2015 because LASSO is not good at capturing the small effects of a large number of variables. In contrast, the estimation of genomic heritability effectively reflects polygene effects. The differences in the characteristics of each estimation method subsequently caused the difference in the adjusted values of *r* for the biomass in 2014 and 2015.

**Specific points to potentially clarify:**

**Line 32: “environmental data was predicted.” Is this in results?**

No, environmental data was not predicted in this study. Because the sentence was misleading, we changed the sentence to “Second, the biomass was predicted from these GP-predicted values and the environmental data using machine learning or crop growth modeling.” (L31-32)

**Line 248. Fourteen traits are mentioned as subjected to genomic prediction, but I did not see a clear summary of these traits.**

Fourteen traits consisted of six intermediate traits and the biomass for two years. The result of the prediction of these traits are shown in fig 5. We added an explanation about the traits (L275).

**Line 322 “Tiller length” but number of tillers in the figure. Please define axes in Fig 3.**

As you mentioned, the trait should be the number of tillers. We corrected the trait name (L349) and apology for the mistake.

**Line 337 Define what is plotted in this figure.. e.g. adjusted line means?**

As you mentioned, we clearly described that the plotted values are “The adjusted mean values of each line” (L361-362).

**Line 402 “An advantage” over what other option?**

It is an advantage over conventional researches of integrated models of GP and CGM. We made the description clearer (L451-452).

**Why specifically is treating parameters as representations of actual crop conditions beneficial?**

One benefit is an easiness to understand the influence of the parameters on the target traits because phenotypic data of intermediate traits are available, as written in the original manuscript (L410-412). Another benefit is the improvement in prediction accuracy in a real datasets because the prediction of complex traits such as yield with an integrated model of GP and CGM was so difficult that no improvement in prediction accuracy had been found in the real datasets [16]. We added a description in the introduction part (L71-77) and the discussion part (L458-463).

**Line 434. The text notes the authors’ approach could save time because biomass is destructive and hard to obtain. This point seems overstated. The intermediate traits themselves do not seem easy to measure. Please clarify.**

As you mentioned, the time-series observation of the intermediate traits is also laborious. We moved the paragraph about the scaling parameter *τ* (L425-435 in the original manuscript) after a paragraph about the measurement of the intermediate traits using high-throughput phenotyping (L522-533). Following the paragraph describing the reduction of the phenotyping cost of the intermediate traits, it is natural to discuss the possibility of reducing the phenotyping cost of destructive sampling.

**In addition, is biomass a useful trait for rice breeders/ others? If so, please state.**

Rice biomass is an important trait for rice breeding, not only by a need of biomass itself to be used as biofuel [31-32], but also as an essential factor to determine grain yield together with harvest index [33-34]. We added the description in the introduction part (L91-92, L95-96) and four references (L668-679).

**This point about the usefulness of the approach was also brought up by the reviewer in terms of validating the model. How much confidence can one have from an analysis of two locations of data?**

It is true that the result will be more confident if we test the model in a larger number of environments. However, we think two environments are not insufficient to prove the ability of the models, as previous researches about an integrated model of GP and CGM also tested in two environments [15-16]. We added a description of this point (L492-494).

**Line 436 “The prediction accuracy of our models was better…” At times, they are equal (Fig 6).**

Your indication is correct. Because this notation is repeated in the result and discussion parts, we erased the sentence.
